# A Systematic Review on Organ-on-a-Chip in PDMS or Hydrogel in Dentistry: An Update of the Literature

**DOI:** 10.3390/gels10020102

**Published:** 2024-01-27

**Authors:** Tania Vanessa Pierfelice, Emira D’Amico, Morena Petrini, Mario Romano, Camillo D’Arcangelo, Ludovico Sbordone, Antonio Barone, Roberto Plebani, Giovanna Iezzi

**Affiliations:** 1 Department of Medical, Oral and Biotechnological Sciences, University G. d’Annunzio of Chieti-Pescara, 66100 Chieti, Italy; emira.damico@unich.it (E.D.); mario.romano@unich.it (M.R.); camillo.darcangelo@unich.it (C.D.); roberto.plebani@unich.it (R.P.); gio.iezzi@unich.it (G.I.); 2 Department of Medicine and Health Sciences “V. Tiberio”, University of Molise, 86100 Campobasso, Italy; ludovico.sbordone@unimol.it; 3 Department of Surgical, Medical, Molecular Pathologies and of the Critical Needs, School of Dentistry, University of Pisa, 56126 Pisa, Italy; antonio.barone@unipi.it; 4 Complex Unit of Stomatology and Oral Surgery, University Hospital of Pisa, 56126 Pisa, Italy

**Keywords:** organ-on-a-chip, oral mucosa-on-a-chip, salivary glands-on-a-chip, oral cancer-on-a-chip, microfluidic devices

## Abstract

Organs-on-a-chip (OoCs) are microfluidic devices constituted by PDMS or hydrogel in which different layers of cells are separated by a semipermeable membrane. This technology can set many parameters, like fluid shear stress, chemical concentration gradient, tissue–organ interface, and cell interaction. The use of these devices in medical research permits the investigation of cell patterning, tissue–material interface, and organ–organ interaction, mimicking the complex structures and microenvironment of human and animal bodies. This technology allows us to reconstitute in vitro complex conditions that recapitulate in vivo environments. One of the main advantages of these systems is that they represent a very realistic model that, in many cases, can replace animal experimentation, eliminating costs and related ethical issues. Organ-on-a-chip can also contain bacteria or cancer cells. This technology could be beneficial in dentistry for testing novel antibacterial substances and biomaterials, performing studies on inflammatory disease, or planning preclinical studies. A significant number of publications and reviews have been published on this topic. Still, to our knowledge, they mainly focus on the materials used for fabrication and the different patterns of the chip applied to the experimentations. This review presents the most recent applications of organ-on-a-chip models in dentistry, starting from the reconstituted dental tissues to their clinical applications and future perspectives.

## 1. Introduction

**Figure 1:**
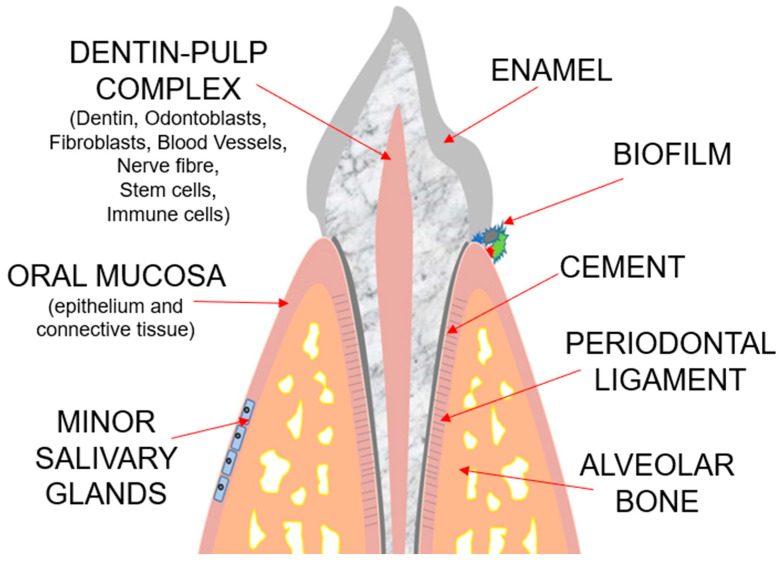
Schematic representation of tooth and supporting tissues.

The drug failure rate in clinical trials remains too high due to the low data translatability to humans. For this reason, in vitro culture models are crucial for identifying drug candidates in preclinical research. Three-dimensional culture models, as well as co-cultures on transwells and organoids, can offer a great advantage over the conventional two-dimensional cell cultures but cannot faithfully reproduce the structure of many organs [1]. In this respect, the organ-on-a-chip technology (OoCT) has revolutionized preclinical research, offering advantages over conventional static 2D and 3D culture models, as it includes shear forces and mechanical strain. Organs-on-a-chip (OoCs) are microfluidic devices constituted by different layers of cells separated by a semipermeable membrane. This technology can set many parameters, like fluid shear stress, chemical concentration gradient, pH variations, and temperature alterations. The use of these devices in medical research allows the investigation of cell patterning, tissue–tissue interface, and organ–organ interaction, mimicking the complex structures of human and animal bodies, thus recapitulating in vitro the complexity of biological microenvironments. One of the main advantages of these systems is that they represent a very realistic model, and with the “FDA Modernization ACT 2.0”, the use of the organ-on-a-chip technology has been approved for drug testing as an alternative to animal models, thus reducing costs and removing ethical issues. The possibility of reproducing a vascular channel and perfusing blood cells, which can migrate from a vascular channel to an inflamed epithelium, makes this technology ideal for studies on inflammation [2]. Monitoring cell movement and migration offers great advantages in investigations about cancer invasiveness, matrix remodeling, and the epithelial-to-mesenchymal transition [3,4]. This technology has been used to faithfully reproduce the function of several tissues and organs, including bronchi, small airways, alveola, the gastrointestinal tract, lymph nodes, blood–brain barrier, liver, vagina, and many others [5,6,7,8,9,10,11]. This accuracy in organ recapitulation permits a high translatability of data from organs-on-chip to humans, so that the OoC has gained great attention in the research field of several diseases, including cystic fibrosis, environmental enteric dysfunction, viral infections, and cancer [5,7,12,13,14]. Moreover, OoCs can be populated by bacteria [12], a microbiota [11], or cancer cells [14]. This technology has also provided insights in the field of dental research, where it was applied for the first time in 2016 to reproduce the complex tooth microenvironments shown in Figure 1 [15,16,17].

Since then, several research groups exploited this technology to investigate and reproduce salivary glands, oral mucosa, and cancer [18,19,20]. In current oral research, one-chamber, multiarray, and parallel-chamber designs were the chip designs usually adopted. The one-chamber chip represents the most common model, consisting of a single culture chamber connected to channels for fluid transport [21]. A multiarray chip is composed of multiple chambers of the same size that are connected by channels and arranged in a matrix. The chambers have the same purpose as the wells for cell culture, and multiarray chips are mostly utilized for high-throughput screening because of the possibility of establishing various conditions in each chamber [22]. In order to explore pathophysiological processes, the parallel-chamber chip is primarily utilized as a scaffold to replicate the natural tissue architecture. This design connects two or more parallel chambers either vertically or horizontally by use of a variety of structures, such as pores, membranes, or tubes [23]. A more complex chip is represented by a serial-chamber design that allows researchers to create a connection between various organ or tissue models through interconnected networks. It is suitable for emulating physiological processes in vitro, like an immune system or a digestive tract [24]. One of the most applied approaches to produce OoCs is lithography, which permits the development of structured surfaces at micrometer and nanometer scales. Microfluidic chip-based models, fabricated via soft lithography and molding, are manufactured with polydimethylsiloxane (PDMS) platforms and present channels and reservoirs that allow fluids to be controlled and manipulated at the microscale level to control experimental conditions (e.g., flow, the concentration of chemical species, rate of chemical reactions) and reduce laboratory costs by decreasing the number of reagents needed for each experiment [25]. Beyond the advantages of OoCs in overcoming the difficulties correlated with ethics and limited throughput, OoCs also permit the minimization of costs with respect to animal experimentation. This technology could be greatly beneficial in dentistry for testing novel antibacterial substances and biomaterials, for performing studies on inflammatory diseases, or for planning preclinical studies. A significant number of publications and reviews have been published on this topic. However, to our knowledge, they mainly focus on the materials used for fabrication and the different patterns of the chip applied to the experimentation. This review presents the most recent applications of organ-on-a-chip models already used in dentistry, starting from the reconstituted dental tissues to their clinical applications.

## 2. Results and Discussion

The main effective results for each tissue of the mouth have been schematically divided and summarized in the tables below each paragraph.

### 2.1. Tooth-on-a-Chip

The tooth represents a unique complex structure in the body that encompasses mineralized and non-mineralized components [26]. The characteristic of enamel is that although it is the hardest tissue in the body, it does not contain cells capable of reproducing or regenerating, so any process that involves the deterioration of this tissue, such as caries, irreparably affects its function and aesthetics. Currently, there is no possibility of regenerating the enamel in vivo, so in the case of loss of this hard tissue, the only option remaining is reconstruction with restorative biomimetic materials. The dentin–pulp complex is a specialized tissue that, in the inner portion, is a soft tissue containing vasculature, nerves, odontoblasts, fibroblasts, stem cells, and an extracellular matrix, and in the external layers is composed of the mineralized dentine [27]. This portion, surrounded by mineralized tissue and therefore incompressible, is particularly susceptible to inflammatory processes of the pulp. The increase in pressure that occurs with the release of inflammatory interleukins is often associated with very painful events for the patient, which often require emergency endodontic treatment, in which the entire vital portion of the tooth is removed, affecting the long-term resistance to trauma of the tooth itself.

Moreover, dental caries cause irreparable functional and aesthetic damage to the entire masticatory system, leading to a deterioration in the quality of life of the patients.

Therefore, the dental field is continuously searching for new forms of prevention and treatment of these pathologies and for new dental-specific regeneration strategies.

**Figure 2:**
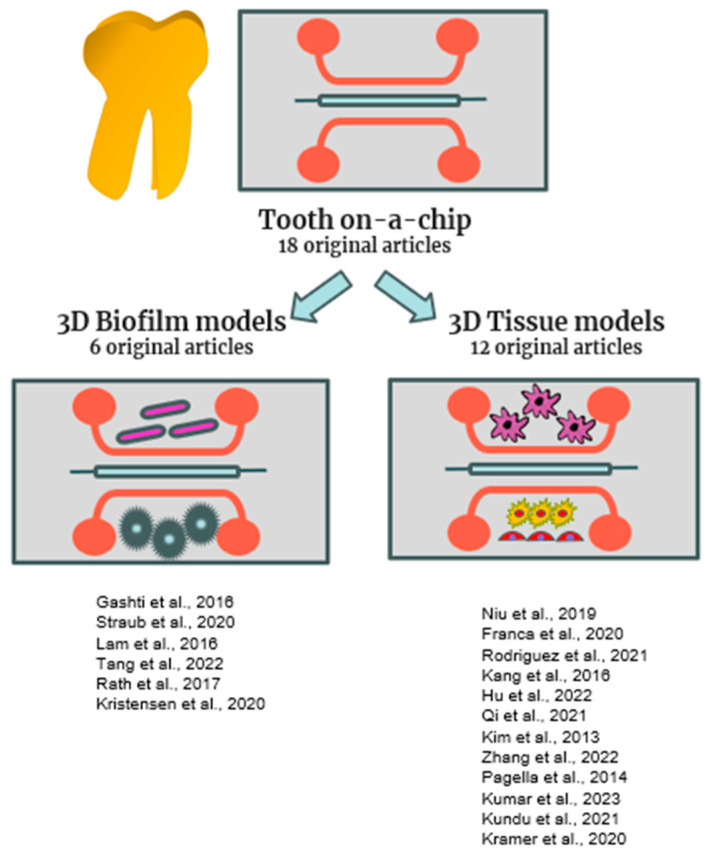
Schematic representation of tooth-on a chip models included in this review [15,16,17,21,28,29,30,31,32,33,34,35,36,37,38,39,40,41].

Most of the studies for novel materials and techniques were primarily tested in in vitro 2D models. However, to test intelligent bioactive materials and drug carriers, there is a need for experimental models able to recapitulate the unique architecture of the non-mineralized tissue (dental pulp) and of the three mineralized tissues (enamel, cementum, dentin). Consequently, before the introduction of tooth-on-a-chip, the only alternative was represented by the animal model. In our review, 18 original articles were selected for the tooth-on-a-chip topic, and they are listed in Figure 2 and Table 1.

Six original manuscripts with only bacteria were included in this section, considering that discovering new therapies to treat bacterial infections remains one of the main objectives of the investigators in this field.

#### 2.1.1. Tooth-on-a-Chip Designs and Materials

The parallel-chamber chip resulted in the most adopted scaffold for mimicking the micro-architecture of dental tissues to examine physiological events. One of the main advantages of the multiple parallel chambers on the chip is the modeling of multifactorial changes in the oral environment, such as temperature and pH fluctuations, bacterial loading, and salivary flow. Strategies for regenerative endodontics are aimed at restoring teeth and prolonging their lifespan by replacing inflamed/necrotic pulp tissues with regenerated pulp-like tissues. However, this purpose requires relevant knowledge of stem cells such as dental pulp stem cells (DPSCs) and periodontal stem cells (PDLs), which ensure the regeneration of dental tissue. Most importantly, there is a need for appropriate modelling platforms to recapitulate the complex microenvironment that sustains the functionality of stem cells [42].

#### 2.1.2. Tooth-on-a-Chip 3D Tissue Models

Kang et al. designed a microfluidic co-culture model for in vitro studies to understand the crosstalk between cells through cytokine gradients by investigating whether secretory factors from human periodontal ligament stem cells (hPDLSCs) and gingival fibroblasts (hGFs) can create an osteogenic environment for stem cells from human exfoliated deciduous teeth (SHED) [16]. In respect to conventional co-culture systems, organ-on-a-chip permits the differentiation between paracrine and juxtacrine effects, which involve multiple cell types. In particular, the parallel-chamber chips are linked in either direction by an assortment of microstructures, such as holes, barriers, and tunnels. Niu et al. developed dentin-on-a-chip for investigating the physiology of odontoblast processes and obtaining an in vitro dentin hypersensitivity model. In this microfluidic model, the presence of 2 μm microchannels demonstrated suitability for observing the growth of odontoblast-related processes [31]. The traditional 2D cell cultures are not appropriate for monitoring the expansion of projections from odontoblast bodies, which in vivo are located in the periphery of pulp, whereas cytoplasmic extensions expand in the direction of the dentin tubules. This parallel-chamber chip allows for the application of a hydrostatic pressure to induce the extension of odontoblast processes toward the microchannels. Microfluidic technology is applied to platforms on chips to investigate tissue interfaces such as hard–soft tissue, bacteria/biofilm–teeth, and biomaterials–tissues. The dentin–pulp interface has been modeled by fabricating various tooth-on-a-chip models for testing the pulp response to different biomaterials [32,33,34]. Franca et al. and Hu et al. both developed a tooth-on-a-chip starting from stem cells from the apical papilla (SCAPs) cultured in odontogenic media, seeded onto a dentin disc that acts as a protective barrier for the pulp. In this way, the resulting pulp should be more resistant to damage than that exposed directly to the test materials. This tooth-on-a-chip has been designed as a testing platform for restorative substances such as phosphoric acid (PA), 2-hydroxyethylmethacrylate (HEMA), silver diamine fluoride (SDF), and Adper-Scotchbond (SB) [32,34], and in respect to 2D cultures, the tooth-on-a-chip replicates the biomaterial–dentin–pulp interface, permitting not only the investigation of cytotoxicity, but also the measurement of the penetration of substances through the dentin [43]. Thus, in the case of pulpitis or pulp necrosis, a potential method for replacing the missing pulp tissue is the transplantation of stem cells, but an inadequate vascularization decreases the vitality of pulp regeneration, and the cone-shaped root canal restricts the blood supply. Since the vascular system arrives to the pulp through the apical foramen at the root apex and proceeds up the root canal to the enlarged pulp chamber of the crown, this aspect remains a challenge for dental tissue engineering [44]. To address this issue, Qi et al. combined a microchannel platform with GelMA hydrogels to support adhesion and proliferation of HUVEC and SCAP cells [35]. This study showed the influence of two parameters, such as the size of taper microchannels and the hydrogel concentrations, on the angiogenic sprouting. The smallest microchannels produce a hypoxic environment that stimulates SCAPs to release angiogenic factors. Kim et al. fabricated a microfluidic chip to form perfusable and functional microvascular networks in a tri-dimensional extracellular matrix construct, by combining the chip with fibrin matrix and collagen I substrate. In this study, researchers co-cultured HUVEC, normal human lung fibroblast, human promyelocytic leukemia cells, and human glioblastoma multiforme cells in a 3D model designed for angiogenesis studies that displays a constant medium flow within the microvascular networks. Thus, this microvascular chip represents a versatile in vitro model applicable for studying vascular biology, but also for investigating the interaction of vascular cells with other cells in a pathophysiology context [36]. Zhang et al. identified the function of semaphorin 4D (Sema4D)-plexin-B1 signaling in the recruitment of SHED as mural cells during vasculature formation in a microfluidic chip combined with a fibrin matrix [37].

Because of the relevance of the nerve system for organs and tissues, nervous cells should be involved in the investigations of pathophysiological processes. However, planar substrates of 2D cultures change the bioelectrical characteristics of neurons, making particularly difficult the modelling of the nervous system. Kundu et al. designed a 3D microelectrode array combined with Matrigel, interfaced with a 3D cellular network, by culturing dorsal root ganglion (DRG) consisting of peripheral sensory neurons and glial Schwann cells. This nerve-on-a-chip demonstrated suitability in an in vitro model for capturing and enhancing the electrical activity of dorsal root ganglion (DRG) cells [40]. Primary dorsal root ganglion (DRG) cells are used to model nerve-on-a-chip for preclinical toxic effect drug testing, since nervous cells are particularly vulnerable to adverse effects of chemotherapies [39,41]. Kumar et al. also fabricated a self-assembly innervated microvasculature-on-a-chip by co-culturing endothelial and DRG cells to study the impact of nervous tissue on the vascular system [39]. The tooth has a peculiar anatomy in which innervation is part of the pulp, as well as vasculature, and plays a key role during the phases of teeth development. Pagella et al. utilized a microfluidic platform to assess co-culture conditions of trigeminal ganglia cells and tooth-derived cells from different stages of tooth development, since this process is intimately correlated with tooth innervation [38]. In this pioneering work, it was demonstrated that trigeminal ganglia and teeth could survive in a microfluidic system for an extended period of time, and that microfluidic co-culture systems are the best method for examining the interaction between neural and dental tissues and the function of innervations in tooth development. 

#### 2.1.3. Tooth-on-a-Chip 3D Biofilm Models

In particular, the multi-chamber chips seem to be particularly suitable for studying biofilms in situ under regulated conditions (i.e., saliva, temperature, shear stress, nutrients). Rodrigues et al. recently utilized the tooth-on-a-chip platform in which the two chambers serve as the pulp and cavity sides, in order to establish the biomaterial–biofilm–dentin interface. This parallel chip allows the investigation of the double responses of both pulp cells and bacteria/biofilm to calcium silicate, by including pH and growth factors variations, in the in vitro model [33]. Thus, microfluidic chips have been employed to perform real-time analyses of bacteria and oral biofilms under dynamic changes in physiological parameters, such as pH and saliva flow [17,28,30]. The microfluidic technology is a powerful tool for studying bacterial adhesion to various dental materials and to assess new antimicrobial treatments under a broad range of parameters that influence the physiology of the mouth [21]. The real-time monitoring with dynamic platforms provides more reliable outcomes than those obtained with cell culture under static parameters. Lam et al. combined sophisticated live imaging and a multiarray chip by applying different gas and sucrose concentrations, to keep track of planktonic cells moving toward a biofilm state [16]. Similarly, Tang et al. designed a multiarray chip to collect specific layers of the produced biofilm under specific environmental parameters and to monitor the mortality rates of *E. coli* during biofilm formation, after antimicrobial treatment [29].

### 2.2. Mucosa-on-a-Chip

The characteristic of soft tissues of the oral cavity is that they are constantly subjected to mechanical and chemical trauma. This tissue, although endowed with regenerative power, can be compromised by the action of chronic inflammation and bacterial and fungal invasions, which can hinder the healing process. Another issue is represented by reactions to foreign bodies that oral mucosa could manifest after exposure to novel materials.

**Figure 3:**
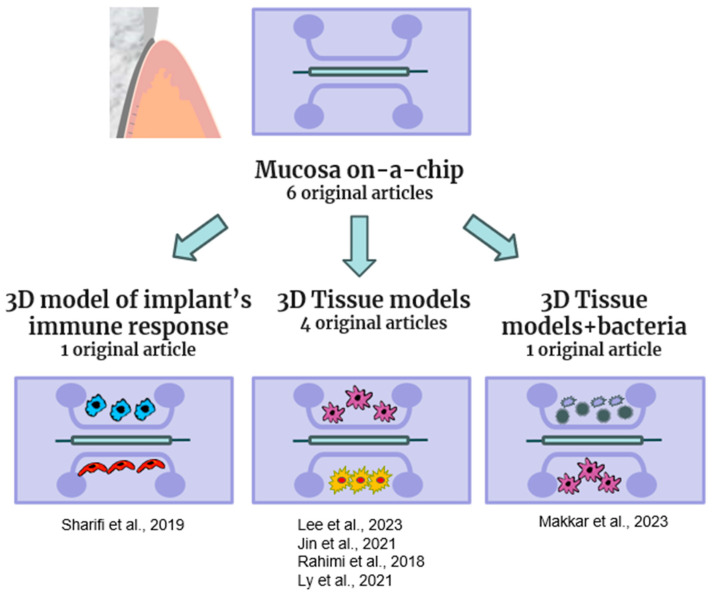
Schematic representation of mucosa-on a chip models included in this review [19,23,47,48,49,50].

Histologically, the oral mucosa comprises three layers: a stratified, thick squamous epithelial layer, basement membrane, and lamina propria, a constructed, vascularized network of the ECM that is residence to fibroblasts and progenitor cells [45]. Thus, to study the response of the oral mucosa, an in vitro model should be able to recapitulate the multiple layers of mucosa tissue. To address this peculiar configuration, the OoC models of the mucosa of other parts of the body have been produced by different authors with different designs, both monolayers and multilayers arranged vertically [25,46]. In our review, six original articles were selected to describe mucosa-on-a-chip, and they are listed in Figure 3 and Table 2.

In terms of oral research, microfluidic platforms have been designed to test the response of the epithelium to bacteria aggression [23], to investigate the epithelial–capillary interface [49] and the epithelial barrier resistance under mechanical stress [48], to study gingival–microbial interaction in periodontal disease [19], and to assess the response of mucosa layers to dental material [23,47,50]. Rahmi et al. designed a microfluidic mucosal model that displayed the apical–basal geometry by seeding keratinocytes in the pores on the side of the central canal where fibroblasts were seeded. The architecture of the tissue construct was assessed through fluorescence staining, which revealed the vertical profile of the cell distribution. Cell viability was evaluated to investigate the response of cells following dental material exposure, and transient epithelial electrical resistance (TEER) was evaluated to investigate the epithelial layer as a barrier following *S. mutans* exposure [23].

The periodontal soft tissue barrier has been designed by co-culturing the key cell components, such as gingival epithelial cells and vascular endothelial cells, on a microfluidic chip platform. This human epithelium–capillary interface was developed through a microdevice with two parallel microchannels separated by a PETE membrane with a thickness of 10 µm [49].

Conventional cultures frequently fail to retain tissue specialization. To overcome this limit, the growth of cells in 3D extracellular matrix gels can improve tissue architecture. However, these approaches continue to fail to recreate structural and mechanical properties of completely organized tissue that are critical to their function. Indeed, mechanical forces given by the extracellular environment affect epithelial cell activity. The 3D oral epi-mucosa device by Lee et al. includes mechanical factors produced by hydrostatic forces, as well as the underlying matrix, to investigate the influence of the microenvironment in regulating adherents and tight junction molecules of the epithelial barrier. This epi-mucosa-on-chip enables the measurement of epithelial integrity, escaping the restrictions linked to animal research and static conditions in 2D systems [48].

Sharifi et al. described an in vitro microfluidic platform that mimics the dynamic effects of circulating immune cells on the implant in foreign body response (FBR) [47]. FBR-on-a-chip (FBROC) can provide a model to interrogate the response to implants, such as biomaterials and engineered tissue constructs, in a physiologically relevant and person-specific way.

### 2.3. Bone-on-a-Chip

Another site that is particularly susceptible to irreversible damage is the alveolar bone. Unlike the basal bone, this develops during tooth eruption, undergoes remodeling during the phases of function, and finally undergoes resorption after the loss of dental elements. Moreover, periodontal disease also causes the resorption of the alveolar bone in the presence of the tooth element. Consequently, edentulous patients usually show different degrees of bone resorption, which needs to be fixed in order to successfully proceed to any oral rehabilitation.

**Figure 4:**
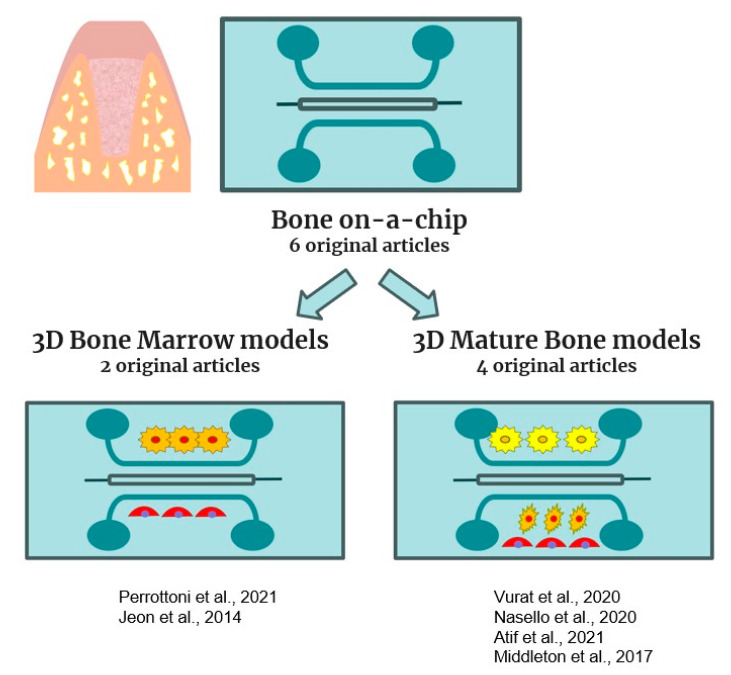
Schematic representation of bone-on-a-chip models included in this review [51,52,53,54,55,56].

Oral bone regeneration represents a topic of increasing interest due to the growing use of dental implants to replace missing teeth. In this respect, potential regulators of bone cell differentiation have been found using a wide range of in vitro screening techniques. These in vitro methods enable us to test possible medications at the cellular level, thereby reducing animal experimentation. However, 2D culture models display various limits in recreating the intricate bone environment, and, among cells that make up bone, osteocytes present unique challenges. In addition to anatomic structure, the highly mineralized nature of bone tissue makes plastic or other planar surfaces an inadequate environmental substitute. In our review, a total of six original articles were selected for bone-on-a-chip and are listed in Figure 4 and Table 3.

Parallel plate flow chambers (PPFCs) are used in most in vitro osteocyte mechano-transduction and cell regulation studies. In these chambers, osteocyte-like cells are exposed to fluid shear stress [51]. However, these culture models lack dynamic and real-time biochemical signaling among the cells involved. Flow-based co-culture has also been accomplished through the development of numerous microfluidic systems. In these devices, cells are embedded in a gel as a model of the extracellular matrix (ECM). However, the gel often decreases the signal transport between the cell populations. In this review, six manuscripts have been included concerning the production of bone-on-a-chip. The proposed models were very different from each other because they aimed to reproduce bone structures at different timings of development (stem cell niches vs. mature bone), or of different types (marrow bone vs. cortical bone) or different sites (interface between periodontal tissue and bone), vascularized or not.

In 2014, Jeon et al. investigated the effects of two endothelial-related factors in a model produced in PDMS, containing endothelial cells (ECs) and bone marrow-derived human mesenchymal stem cells (BM-hMSCs) [51]. This 3D model represents a significant advancement in producing microvessels nearer to physiology than endothelialized micro-networks generated within 3D gels or spheroids.

In 2021, Perottoni et al. proposed a model to replicate the microenvironmental conditions of the perivascular niches, constituted by microchambers in polycarbonate (PC)-containing bone marrow-derived human MSCs (h-MSCs). This niche-on-a-chip setup permits the support of a transient microenvironment with a fluctuating spatial distribution of oxygen tension that is crucial to the perivascular stem cell niche [52]. This platform represents a model that allows the profiling of stem cell metabolism, and it would be a reliable device for drug screening and disease modelling.

In 2021, Atif et al. proposed a PDMS model containing pre-osteoblast-like MC3T3-E1 cells cultured in a platform with hydroxyapatite (HA). Different flow rates were applied to this HA-on-a-chip to test the biological response to this material under conditions nearer to in vivo physiology [55].

In 2020, Nasello et al. produced a micro-engineered platform to study osteoblast differentiation into osteocytes. This bone-on-a-chip combined microfluidic technology with a 3D fibrous collagen matrix to produce a more reliable model that replicates the maturation of osteoblasts into osteocytes and matrix mineralization [54]. The bone-on-a-chip described in this work might offer the minimal functional human osteoblast microenvironment to construct patient-specific bone models for investigating the effects of alternative therapies.

In 2020, Vurat et al. developed a microfluidic platform containing gelatine methacryloyl (Gel-MA) and hydroxyapatite–magnetic iron oxide nanoparticles, to cultivate periodontal ligament fibroblasts (hPDLFs) and osteoblasts (hOBs) [53]. This double-layered 3D-bioprinted microtissue model of the human periodontal ligament–alveolar bone interface has the potential to be a preclinical platform for evaluating drug effects on periodontal sites.

In 2017, Middleton et al. established a co-culture of osteoclast precursors and osteocytes under different flow rates in a microfluidic model without the addition of any gel [56]. This platform was used to study the influence of osteocytes on osteoclasts under various conditions, and it can potentially be a tool to investigate the crosstalk between bone cells in a pathology status. Although bone cell function, bone regeneration, vasculature, and response to some materials have been investigated by current bone-on-a-chip technologies, no particular chips have been introduced to look into the maxillary and mandibular bones.

### 2.4. Oral Cancer-on-a-Chip

Oral cancer represents 90% of malignant cancer of the head and neck [57] and accounted for approximately 180,000 deaths worldwide in 2018 (1.9% of total cancer cases). It is among the top 15 most common cancers worldwide [58] and is characterized by high morbidity and mortality. Organ-on-a-chip has emerged as a breakthrough in cancer research as it provides a dynamic platform to simulate tumor growth and progression in a chip. It also has been successfully employed in recent years, especially for diagnostics and prognosis purposes in cancer research. Regmi S et al. provide an overview of microfluidics and organ-on-a-chip technology, reviewing their historical development, physics of fluid flow, and application in oncology [59]. A first approach was developed by Nguyen et al., who applied the 3D-printed technique to manufacture droplets of varying size with variable frequencies. This droplet device permits the generation under the control of Ca-alginate microspheres containing A549 cells, which may be employed for tumor spheroid production [60].

**Figure 5:**
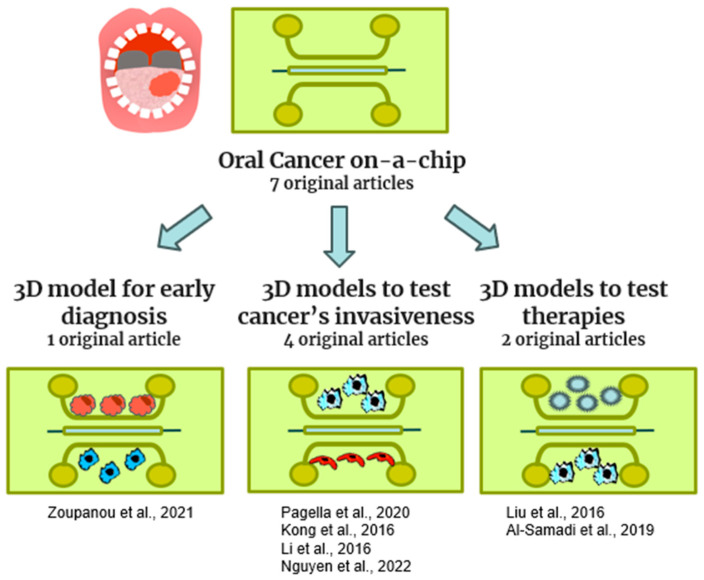
Schematic representation of cancer-on-a-chip models included in this review [20,60,61,62,63,64,65].

In our review, a total of seven original articles were selected for oral cancer-on-a-chip, and they are listed in Figure 5 and Table 4.

The models proposed reproduced oral cancer in different sites of the oral cavity, like the tongue, salivary glands, and bone, and the metastasis of primitive extraoral cancers in the mouth.

Al-Samadi et al. produced a 3D tongue cancer model characterized by a parallel PDMS chip cultured with a tongue cancer cell line (HSC-3) and hMNC (monocytes) [65]. Their objective was to test the efficacy of immunotherapy on this pathology in order to develop personalized medicine therapeutics. However, early diagnosis is important for the success of the treatment and for survival [65]. In 2021, Zoupanou et al. proposed a serpentine model in PMMA containing human oral cavity squamous cell carcinoma (OECM-1) in conjunction with Jurkart cells (t-cell leukemia) to produce a tool for early diagnosis of oral squamous carcinoma. The invasiveness of oral cancer is very strong; therefore, the study of the mechanisms that promote this phenomenon could represent useful support for the management of this pathology [61]. Pagella et al. proposed a 3D microfluidic device with a co-culture of ameloblastoma cells and trigeminal ganglia cells to study cell–cell interactions [62].

The other three studies proposed organ-on-a-chip models containing adenoid cystic carcinoma cells of the salivary glands. Kong et al. proposed a biomimetic microfluidic model to study cancer metastasis using primary cells isolated from different organs. They demonstrated that OoCs are useful to expand the capabilities of traditional cell culture models, using a low-cost, time-saving, and rapid alternative to animal models. In addition, the metastasis process is promoted by the growth of new blood vessels; thus, anti-angiogenic agents could offer novel therapeutic opportunities in oral cancer [63]. Liu et al. developed a microfluidic model to study the angiogenic potential of salivary gland adenoid cystic carcinoma (ACC) and oral squamous cell carcinoma and to evaluate the effects of antiangiogenetic drugs. Carcinoma-associated fibroblasts (CAFs) promote tumor invasion and metastasis, although the role of CAF is poorly understood [20]. Li et al. isolated CAFs from ACC that were co-cultured with ACC cells in a microfluidic device in order to study the invasion capability of CAFs. This capability was evaluated by the analysis of matrix metalloproteinase expression and by wound healing and cell invasion assays [64].

### 2.5. Salivary Glands-on-a-Chip

**Figure 6:**
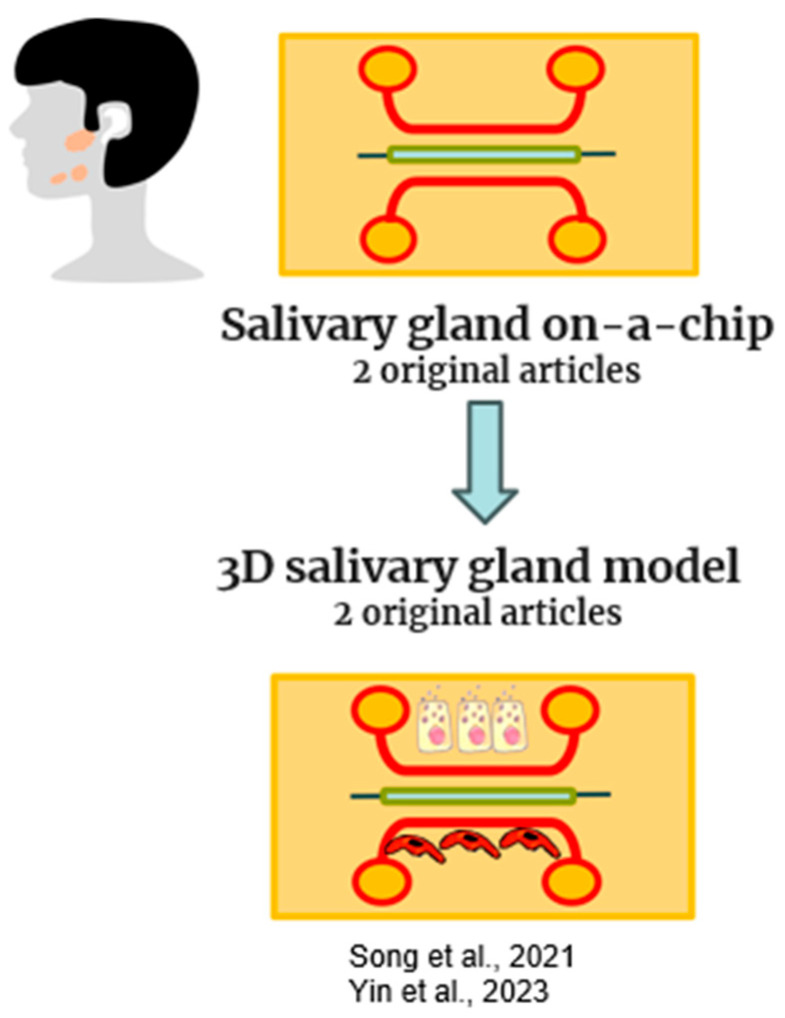
Schematic representation of salivary gland-on-a-chip models included in this review [18,70].

The greatest difficulty in the 2D culture of salivary gland cells is the rapid loss of secretory function observed in these cells. Saliva is necessary for good oral mucosa and dental health, and a reduction in saliva production, known as xerostomia, has severe effects on enzymatic digestion, dentition, bacteriostatic functions, and fundamental tasks, including eating and speaking. The salivary glands display a highly branched and secretory architecture, in which differentiated cell types create epithelial ducts and acini. The first 3D culture model for recapitulating salivary gland structure included scaffold-based hydrogels that allow salivary gland cells to generate spheroids capable of differentiating into acinar-like architecture [66,67]. More recently, a higher expression of salivary acinar, ductal, and tight junction markers was obtained in salivary gland cells seeded in a microwell culture than in those grown in 2D and matrigel-3D cultures [68,69]. However, these 3D culture approaches fail to obtain spatial control, particularly when addressing the branching architecture of native salivary glands and the extremely thin epithelial layers of the ducts and acini. Microfluidics technologies, on the other hand, provide superior management of both the spatial and temporal distribution of biomaterials, by obtaining a controlled shear stress. In our review, two original articles were selected for salivary gland-on-a-chip, and they are listed in Figure 6 and Table 5.

Yin et al. recently used a coaxial microfluidics (CMF)-based bioprinter to print hydrogel fibers and tubes with various dimensions to replicate the size and features of salivary epithelia. In particular, solid alginate fibers were printed to outline the branching structure of salivary glands. The alginate tubes, which have extremely thin walls and an open lumen, fit within the dimensions of salivary epithelial layers. Immunocytochemistry characterizations revealed that stem cells (hS/PCs) retain stemness markers in 3D bioprinted tubes for up to 15 days [18]. Song et al. combined the supportive microenvironment supplied by matrix metalloproteinase (MMP)-degradable PEG hydrogels with microbubble array technology to create a modular salivary gland tissue chip platform. In this salivary-gland-on-a-chip, salivary cells enclosed in the MMP-degradable PEG hydrogels remained viable, expressed specific markers, and released salivary proteins in response to calcium signaling agonists. The salivary-gland-on-a-chip served to investigate radiosensitivity and radiation damage reduction using a radioprotective agent [70]. Considering that the scarcity of in vitro models that replicate salivary gland function is hampering the progress in the development of novel treatment options, this in vitro model has the potential to mimic salivary gland activity and may allow high-throughput drug screening.

### 2.6. Future Trends

Most current OoCs are developed to emulate specific tissue components or certain functions of in vivo organs, such as renal proximal tubules, kidney glomeruli, small lung airways, and lung alveoli. However, the final aim is to integrate numerous organs into a single chip and build a more complex multi-organ chip model, finally achieving a “Human-on-a-chip”. Due to limited technologies, reconstructing whole organs with intact structures and functions in vitro is still impossible. This aspect is particularly critical for the dental sector, considering that diseases in other parts of the body often arise in the oral compartment, for example, different oral microorganisms are involved in different disorders outside the mouth.

Currently, the cost of manufacturing OoCs is relatively expensive, and their widespread use requires low-cost and large-scale manufacturing in a repeatable and standardized manner. Standardization is a requisite to transform organ-on-a-chip technology into high-throughput organ-on-a-chip technology, with the final aim of using this technology to expedite the screening process in drug development, prevention, and early diagnosis. Regulatory agencies should also lay down guidelines for validating organ-on-a-chip technology for various potential applications, including disease model development. Parallelization of models, a standardized and scalable platform, validation, automation, and online data analysis are some of the crucial elements that should be included in high-throughput organ-on-a-chip models. 

The preservation of OoC function over long periods of time represents another goal to achieve in the future. To date, dynamic models that permit the connection of fluid channels have been developed; however, they do not allow long-term culture maintenance. 

Currently, most OoCs are fabricated with PDMS using soft lithography, but the reproducibility of fabrication is questionable for the large-scale production of devices for the market. An alternative method could be represented by 3D bioprinting, a promising technique for fabricating OoC devices able to produce sophisticated tissue architectures, complex scaffolds, or device templates with high fidelity and controllability.

In the future, OoC platforms could be developed using patient-derived materials, such as patient tissue, decellularized ECM, and other biological materials, from the perspective of personalized precision medicine. 

In the present revision, all OoCs are still far from representing the full complexity of the structures of the oral cavity. Currently, the proposed models are only stylized representations of natural structures. In order to increase its clinical potential, it will therefore be important to develop models that are increasingly closer to reality, in which hard tissues interface with soft tissues in a dynamic environment. OoCs in dentistry should be produced not only with all mineralized and soft tissues, as in real teeth, but also including an environment like the oral cavity, using devices that could replicate the masticatory cycles, like chewing machines. Only in this way could these models reproduce the in vivo conditions and reach full transferability to clinical application.

## 3. Conclusions

In conclusion, the potential utility of organ-on-a-chip lies in its use in the pharmaceutical industry, in identifying novel biomarkers, in elucidating the pathogenesis of diseases and the metabolic activities of human cells, and in the development of personalized precision medicine.

Microfluidic technology is used in various in vitro setups to investigate physiological and pathological processes and study the host response to external agents. Although this technology is not yet available in clinics, encouraging in vitro results should help to quickly gain traction, and despite the fact that microfluidic platforms are being adopted in many medical fields, they are relatively new in dentistry. Organ-on-a-chip would fill a gap in preclinical research; however, most of the platforms related to the oral cavity are primordial models and still lack the complexity of the oral tissues. While representing tissues better than 2D cultures, these models often do not include more than two cell typologies and often do not include cells derived from the oral cavity itself. Literature on tooth-on-a-chip primarily focuses on investigations concerning bacteria, the interface with biomaterials, and studies on the pulp (micro-vascularization and innervation) and/or germination, often developed only to test drugs. Mucosa is the most properly represented oral tissue, developed by culturing cells derived from the oral cavity, while to date, alveolar bone-on-a-chip is still lacking. Among current bones-on-a-chip, just one was developed by including cells from oral tissues. Thus, there is a gap to be filled considering the number of disorders that involve alveolar bone. Oral cancer is another topic poorly addressed compared to cancers arising in other body districts, despite the fact that mouth cancer is highly diffused worldwide. A 3D in vitro model could be helpful to improve diagnostics and therapeutics. A salivary gland model would also be relevant to test therapies for the restoration of damaged glands by anticancer therapy.

Thus, the ideal future models of tooth-on-a-chip should comprehend the mineralized and non-mineralized dental tissues as well as the cellular components present in the oral cavity, to reconstitute the environment where the teeth are constantly subjected to mechanical, physical, and chemical trauma.

## 4. Materials and Methods

### 4.1. Inclusion Criteria

The protocol of this review has been developed according to the PRISMA (Preferred Reporting Items for Systematic Review and Meta-Analyses) statement [71,72].

The systematic review was designed to answer the following focused questions:

- Why are OoCs potentially important for dental clinical practice?
- What are the OoCs’ current and future applications in dentistry?

We conducted a search strategy with the following keywords:

“organ-on-a-chip” OR “microdevice” OR “Microfluid” OR “tooth-on-a-chip”) AND (“oral mucosa” OR “oral” OR “tooth” OR “dental” OR “caries” OR “pulp” OR “salivary glands” OR “tongue” OR “periodontal” OR “enamel” OR “dentin” OR “cementum” OR “roots” OR “decays” OR “alveolar bone” OR “gums” OR “teeth” OR “pulpitis”).

The search has been conducted on different electronic databases, including Scopus, Pubmed, and Web of science.

Filters used were English language.

The presence of duplicates was assessed through Mendeley software 2.109.0. Two independent expert researchers (MP and RP) performed the title and abtract screening and then the full-text analysis. All types of manuscripts were included in the electronic search, but the reviews included in the full-text analysis were consulted only to perform a further hand search and to find other original manuscripts for the qualitative analysis. The full texts of the included manuscripts were analyzed, and the references were grouped according to the clinical application of the organ-on-a-chip developed: tooth, mucosa, bone, salivary glands, and carcinoma. Data were extrapolated in tables for qualitative analyses, accordingly to Huang et al., 2023 [46].

**Figure 7:**
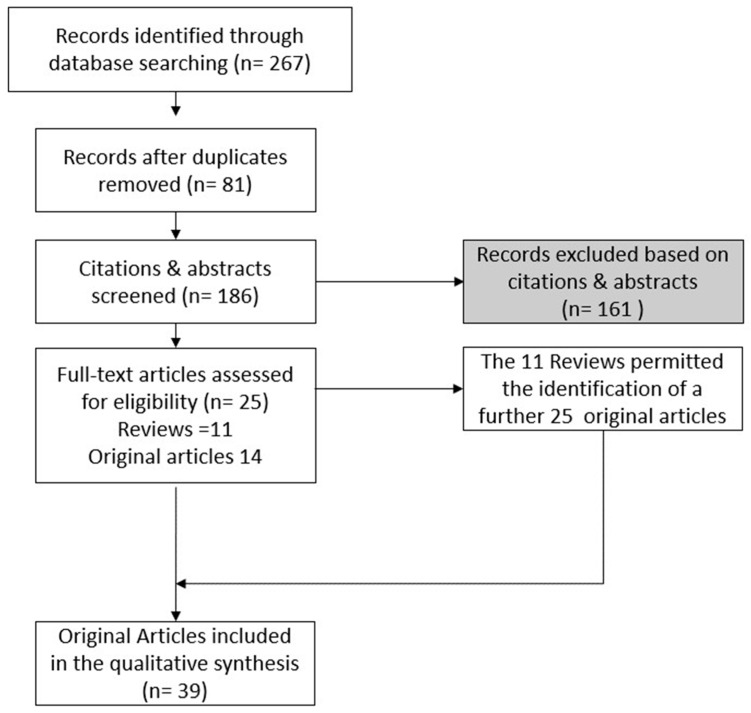
Flow chart of methodologies to obtain the revised original manuscripts.

In total, 39 original manuscripts were included in the qualitative analysis (Figure 7).

### 4.2. Selection of the Manuscripts

**Figure 8:**
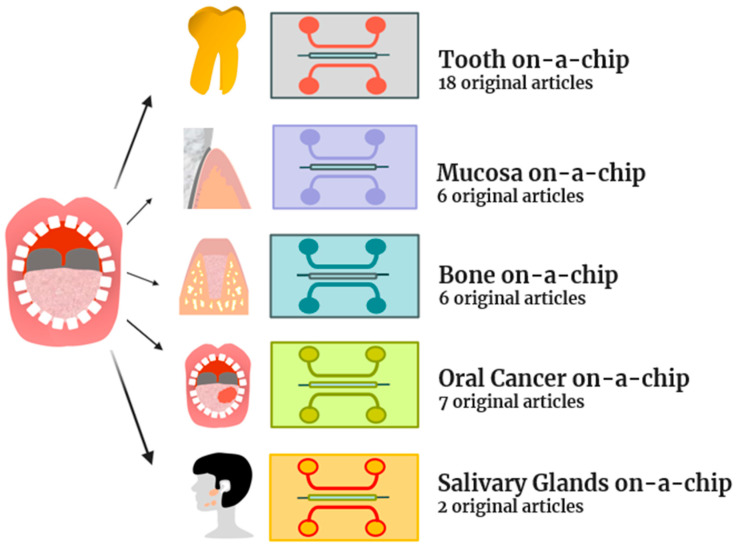
The topics of the 39 original articles included in the review.

The electronic search identified 267 titles, 116 manuscripts on Scopus, 75 on Pubmed, and 76 on Web of Science. After duplicate removal, 186 titles were included for title and abstract screening. Two reviewers (MP and RP) conducted the title and abstract revision. Any discrepancies were overcome through the discussion of the two auditors, which led to a common solution. A total of 25 manuscripts (14 original articles and 11 reviews) were included in the full-text analysis. Of these, the 11 reviews included and listed in Table 6 were used for an additional hand search that permitted the inclusion of another 25 original articles. Thus, in total, 39 original manuscripts were included, and they are listed in Table 7. As shown in Figure 8, the 39 original manuscripts described original novel models of tooth-on-a-chip (18), mucosa-on-a-chip (6), bone-on-a-chip (6), oral cancer-on-a-chip (7), and salivary glands-on-a-chip (2).

The 11 review articles included in the full-text analysis are reported in Table 6.

The 39 original studies included in the qualitative analysis are reported in Table 7.

## Figures and Tables

**Table 1:** This table includes original manuscripts that describe tooth-on-a-chip models.

Reference	Organ-on-a-Chip	Aim	Chip Design	Material	Cell Type	Applied Methods	Future Applications
Gashti et al. (2016) [17]	Microfluidic platform of oral biofilm	To study oral biofilm under different conditions	One chamber	PDMS	*S. salivarius*	pH measurements, and confocal fluorescence (CLSM)	To test localized acidification in the oral biofilm
Straub et al. (2020) [28]	Microfluidic platform of oral biofilm	To obtain real-time observation of bacterial adhesion and biofilm formation	Parallel	PDMS	*E. coli*	Single-cell tracking analysis	To study bacterial and surface interactions. To establish a tool for studying the in situ activity of antimicrobial agents against surface-associated bacteria and cells in a biofilm.
Lam et al. (2016) [16]	Artificial teeth	To study the bacterial growth under a matrix of different combinations of microenvironmental factors	Multi-array	PDMS	*Streptococci species, F. nucleatum*	CLSM, live dead, bacterial assay	To perform biofilm analysis and to test anti-biofilm agents
Tang et al. (2022) [29]	Microfluidic platform of oral biofilm	To develop a model for studying the antibiotic resistance dynamics	Multi-array	PDMS	*E. coli*	In situ staining, determination of MIC	Applications of this device in many areas of microbiology where biofilms are common
Rath et al. (2017) [21]	Microfluidic platform of oral biofilm	To study biofilm formation on titanium surfaces	One chamber	Polyaryletherketone (PAEK) + titanium	*S. gordonii, S. salivarius, S. oralis, P. gingivalis, A. actinomycetemcomitans*	Live dead	To optimize the study of the formation of oral multispecies biofilms. To investigate the influence of different flow velocities, nutrient concentrations, and substrata on the biofilm formation
Kristensen et al. (2020) [30]	Microfluidic platform of oral biofilm	To develop a flow cell model for bacterial studies	One chamber	Resin by 3D printing	Biofilm from healthy volunteers	pH, CLSM	To study the effect of different flow rates on pH in biofilms of different age in a larger subject group to understand the caries process
Niu et al. (2019) [31]	Microstructures of dentin tubules-on-a-chip	To study the physiology of dental pulp	Parallel	PDMS + collagen	Odontoblast cells; Mouse Dental Papilla Cell (MDPC-23)	CLSM	A tool for investigating the mechanisms of dental diseases and developing effective treatments
França et al. (2020) [32]	Tooth-on-a-chip	Dentine/pulp model for Cell testing of HEMA, phosphoric acid, scotch bond	parallel	PDMS + dentin	Stem Cells From the Apical Papilla (SCAP)	Live dead, cytotoxicity, gelatinolytic activity assay, metabolic activity assay	To test pulp response to biomaterials
Rodrigues et al. (2021) [33]	Dental pulp-on-a-chip	Dentine/pulp model for testing calcium silicate cements	parallel	PDMS + Dentin + Collagen 1	Human dental pulp stem cells (hDPSC), *S. mutans*	CLSM, ELISA, pH, Live dead	To test pulp response to biomaterials and to bacteria
Kang et al. (2016) [15]	Tooth-on-a-chip	To investigate the mineralization of SHED cells in an oral cell niche	parallel	PDMS	human gingival fibroblasts (hGFs), periodontal ligament stem cells (hPDLSCs), human exfoliated deciduous teeth (SHED)	Alizarin Red Staining (ARS), RT-PCR	A model to examine the effects of ecretory factors from various oral cells on pulp stem cells
Hu et al. (2022) [34]	Tooth-on-a-chip	Dentine/pulp model for testing silver diamine fluoride	one chamber	Polymethyl methacrylate (PMMA) + Dentin	DPSCs (dental pulp cells), hGFs, human keratinocytes (OKF6/TERT1)	MTT, mucosal corrosion test	To test pulp response to biomaterials
Qi et al. (2021) [35]	Endothelized Fluidic microchannels	To test angiogenic sprouting	one chamber	Gelatin metacryloyl (GelMa) hydrogel	SCAP, Human umbelical vein endothelial cells (HUVECss)	CLSM	Root canal model for endodontic regeneration
Kim S et al. (2013) [36]	Microvascular networks-on-a-chip	To form perfusable and functional microvascular networks in 3D ECM constructs.	parallel	PDMS + fibrin matrix + collagen I	HUVEC, normal human lung fibroblast, Human promyelocytic leukemia cells, Human glioblastoma multiforme cells	CLSM, Measurement of vessel permeability, Fluid perfusion experiments, analysis of endothelial NO synthesis	A versatile in vitro model for the fundamental study of vascular biology and vascularized micro-organs or human disease models for drug discovery
Zhang et al. (2022) [37]	Vascularized dental pulp-on-a-chip	To study the SHED cell recruitment during angiogenesis	parallel	Chip from AIM Biotech + fibrin gel	HUVEC, SHED	CLSM, Western Blot (WB), Optical microscope (OP), ELISA, RT-PCR, Vessel permeability assay	A model to study vascular development
Pagella et al. (2014) [38]	Dental pulp nervous system-on-a-chip	To study whether trigeminal ganglia and teeth can survive for long periods of time in microfluidic devices	parallel	PDMS + poly-D-lysine and laminin	Trigeminal ganglia, Incisor tooth germs	Immunohistochemistry (IHC), CLSM	To study tooth germ formation kinetics
Kumar et al. (2023) [39]	Innervated Vasculature-on-a-chip	To develop a microfluidic model of innervated vasculature to study the interface between sensory neurons and vasculature	one chamber	PDMS + Poly-D-Lysine	HUVEC, murin and human primary dorsal root ganglion neurons (DRGs)	CLSM	To develop innervated micro-physiological models.
Kundu et al. (2021) [40]	Nerve-on-a-chip	To design a 3D microelectrode to obtain a nerve-on-a-chip	multi-arrays	Photopolymer clear resin (3D printed) layer of SiO_2_	DRG	OP, scanning electron microscopy (SEM), Fourier-transform infrared spectroscopy (FTIR), propidium iodide and calcein for viability	To simulate “disease in a dish”
Kramer et al. (2020) [41]	Nerve-on-a-chip	To develop a rat nerve-on-a-chip to demonstrate its potential as a preclinical assay for screening for drug-induced nerve dysfunction	one chamber	Polymerized hydrogel	DRG	Compound action potentials (CAPs), Nerve conduction velocity (NCV), LDH Cytotoxicity Assay, Cell Counting Kit-8 (CCK-8), Light microscopy (LM), transmission electron microscopy (TEM), Mitochondrial membrane potential assay, IHC	To develop an effective tool for screening potentially harmful drugs and other neuropathy-inducing agents

**Table 2:** This table includes original manuscripts that describe mucosa-on-chip models.

Reference	Organ-on-a-Chip	Aim	Chip Design	Material	Cell Type	Applied Methods	Future Applications
Lee et al. (2023) [48]	3D Oral Epi-mucosa platform	To investigate the epithelial barrier function in conditions of mechanical stress	one chamber	PDMS + collagen gel	Human immortalized gingival keratinocyte (hIGK) cells	Scanning Electron Microscope, Mechanical Testing, Epithelial Permeability Measurement, CLSM, Staining, RT-PCR and RT-qPCR Analysis	A model to elucidate new pathways involved in oral diseases
Sharifi et al. (2020) [47]	Foreign Body Response-on-a-chip (FBROC)	To investigate the response of circulating immune cells to the implants	one chamber	PDMS + PMMA + GelMA	HUVEC, human monocytes THP-1 (human leukemia monocytic cell line)	3D conducted computational fluid dynamics (CFD) simulations, ELISA, CLSM	A model to interrogate the response to biomaterials
Makkar et al. (2023) [19]	Gingival crevice-on-chip	To study host–microbial interaction in periodontal disease	parallel	PDMS + fibrin gel	Gingival fibroblasts (hGFs), *S. oralis*, *F. nucleatum*	Cytotoxicity, Live dead, ELISA, CLSM	In vitro model of periodontal pocket including more oral cells
Jin et al. (2021) [49]	Periodontal soft tissue-on-a-chip	To develop a model of epithelial–capillary interface	parallel	PDMS + PETE membrane	Huvec, Gingival epithelial cells (hGEs)	CLSM	In vitro model of periodontal pocket to study diseases related to periodontitis
Rahimi et al. (2018) [23]	Oral mucosa-on-a-chip	To investigate the response to bacteria and dental materials (HEMA)	parallel	PDMS + collagen I	Human gingival Keratinocytes (hGKs), hGFs, *S. mutans*	CLSM, Live dead, impedance spectrometer	For testing the response to biomaterials and bacteria by incorporating the patient’s immune cells
Ly et al. (2021) [50]	Oral mucosa-on-a-chip	To study the response of gingiva to dental biomaterials (HEMA at different concentrations)	parallel	PDMS + collagen I	Human gingival Keratinocytes (hGKs), hGFs	CLSM, Live dead	Evaluation of tissue response to various biomaterials

**Table 3:** This table includes original manuscripts that describe bone-on-a-chip models.

Reference	Organ-on-a-Chip	Aim	Chip Design	Material	Cell Type	Applied Methods	Future Applications
Vurat et al. (2020) [53]	Periodontal ligament–alveolar bone 3D bioprinted	To develop a multicellular 3D-bioprinted microtissue model of human periodontal ligament–alveolar bone bio-interface	one chamber	PDMS + Methacrylated gelatin (Gel-MA) + hydroxyapatite–magnetic iron oxide nanoparticles	Human periondal ligament fibroblasts (hPDLFs), osteoblasts (hOBs)	Rheology, ATR-FTIR analysis, Thermogravimetric analysis, Mechanical analysis, SEM, Swelling testing, Alamar blue assay, Live/dead cell staining, CSLM, histochemical staining (PAS)	In vitro model to study the potential effects of drugs
Perottoni et al. (2021) [52]	Perivascular niche-on-a-chip	To develop and validate a miniaturized platform for profiling stem cell metabolism in a niche-on-a-chip	parallel	Oxygen-impermeable polycarbonate (PC)	Bone marrow-derived human MSCs (h-MSCs)	Computational fluid dynamic (CFD) analysis, Multiphoton quantitative intracellular oxygen imaging, Two-photon fluorescence lifetime imaging microscopy (2P-FLIM)	To provide a reliable tool for disease modelling and drug screening
Nasello et al. (2020) [54]	Bone-on-a-chip	To develop a bone-on-a-chip device to study the osteoblast differentiation into osteocytes	parallel	PDMS + hydrogel	hOB	CSLM, inverted brightfield microscope, calcein green staining, extracellular ALP activity.	To develop patient-specific bone models to study the individual osteogenic potential and the effect of alternative therapies
Jeon et al. (2014) [51]	Microvascular network-on-chip	To generate a functional, perfusable 3D human microvascular network	parallel	PDMS + hydrogel	Endothelial stem cells (ECs), bone marrow-derived human mesenchymal stem cells (BM-hMSCs)	CSLM, Vessel permeability	To test diffusion and effects of therapeutics in complex microenvironments
Atif et al. (2021) [55]	Hydroxyapatite (HA)-on-chip	To integrate HA in a microfluidic platform and to assess the behavior of pre-osteoblast	parallel	PDMS + biomimetic hydroxyapatite	Mouse osteoblasts (MC3T3-E1)	Cell viability, LDH, ALP assay	A tool to evaluate the biological properties of biomaterials
Middleton et al. (2017) [56]	Bone-on-a-chip	To study the cross-talk between bone cells under different flow conditions	parallel	Not specified	Osteoclast precursors (RAW264.7), osteocytes (MLO-Y4)	CSLM	A tool for investigating bone response to different stimuli

**Table 4:** This table includes original manuscripts that describe oral cancer-on-a-chip models.

Reference	Organ-on-a-Chip	Aim	Chip Design	Material	Cell Type	Applied Methods	Future Applications
Pagella et al. (2020) [62]	Innervated ameloblastoma-on-a-chip	To study the interaction between ameloblastoma cells and trigeminal ganglia cells	Parallel	PDMS + laminin	ameloblastoma cells, Trigeminal Ganglia cells from mouse	CLSM	In vitro model to study the invasiveness of cancer cells
Kong et al. (2016) [63]	Organ metastasis of circulating tumor cells-on-a-chip	To assess the potential of breast and salivary gland cancer cells to metastasize to the lung	parallel	PDMS + collagen I	HUVEC, MCF-7, and MBA-MD-231 (breast cancer cells), salivary gland adenoid cystic carcinoma cell line (ACC-M), Primary murine pulmonary cells, Primary murine hepatocytes	ELISA, CLSM, and Flow cytometry	An in vitro model to predict the metastatic capabilities of tumor circulating cells, and to rapidly screen possible anti-metastatic drugs.
Liu et al. (2016) [20]	Tumor-induced angiogenesis-on-a-chip	To investigate metastasis and invasiveness processes in oral cancer	Parallel	PDMS	HUVEC, oral squamous cell carcinoma (UM-SCC6), Salivary gland adenoid cystic carcinoma (ACC-M)	CLSM,	An in vitro model to test future anti-cancer and antiangiogenic drugs
Zoupanou et al. (2021) [61]	Plug-and-play device	To develop a device for early screening of oral squamous cell carcinoma	Serpentine	PMMA functionalized with O_2_ plasma	Jurkart cells (t-cell leukemia), human oral cavity squamous cell carcinoma (OECM-1)	Tests to distinguish cancer cells from blood cells	Early diagnosis of oral carcinoma
Li et al. (2016) [64]	Adenoid cystic carcinoma (ACC) platform	To study the role of Carcinoma-associated fibroblasts (CAFs) in the progression of adenoid cystic carcinoma (ACC)	Parallel	PDMS	CAF from (ACC) patients, human salivary adenoid cystic carcinoma cell line (SACC83), metastatic lung cells (LM)	Optical microscope, cell invasion and migration assay, wound healing, CLSM	A model to study ACC progression
Nguyen et al. (2022) [60]	A 3D-printed size-tunable flow-focusing droplet microdevice	To develop a droplet device that permits the generation under control, Ca-alginate microspheres containing tumor cells		Resin by 3D printing	A549 adenocarcinomic human alveolar basal epithelial cells	CLSM	A tool for tumor spheroid production
Al-Samadi et al. (2019) [65]	Tongue cancer-on-a-chip	To test the efficacy of immunotherapy	parallel	PDMS + myogelfibrin	tongue cancer cell line (HSC-3), and monocytes (hMNC)	CLSM	A 3D model to study novel therapies for tongue cancer

**Table 5:** This table includes original manuscripts that describe salivary-gland-on-a-chip models.

Reference	Organ-on-a-Chip	Aim	Chip Design	Material	Cell Type	Applied Methods	Future Applications
Song et al. (2021) [70]	Salivary gland model	To develop functional tissue mimetics for mouse and human salivary glands	multi-arrays	PDMS + Hydrogel	Mouse acinar cell clusters and intercalated ducts (AIDUCs)	LIVE/DEAD, RT-PCR, CMLS, Calcium signalling assay, amylase activity	To provide a tool for mechanistic studies and for clinically predictive screening assays
Yin et al. (2023) [18]	Salivary gland model	To produce with a 3D printer a Microfluidic device cell-laden microfibers and microtubes for salivary gland tissue engineering	multi-arrays	Alginate hydrogel	human Salivary Stem progenitor cells (hS/PCs)	LIVE/DEAD, calcein, Ethidium Homodimer-III, different imaging techniques, Immunochemistry	To produce a model that mimics salivary glands

**Table 6:** Review articles included in the full-text analysis.

1	Huang C, Sanaei F, Verdurmen WPR, Yang F, Ji W, Walboomers XF. The Application of Organs-on-a-Chip in Dental, Oral, and Craniofacial Research. J Dent Res. 2023 Apr;102(4):364–375. https://doi.org/10.1177/00220345221145555. Epub 2023 Feb 1. PMID: 36726271; PMCID: PMC10031637. [46]	Review
2	Tiozzo-Lyon P, Andrade M, Leiva-Sabadini C, Morales J, Olivares A, Ravasio A, Aguayo S. Microfabrication approaches for oral research and clinical dentistry Front. Dent. Med, 09 March 2023 Sec. Dental Materials. Volume 4—2023 | https://doi.org/10.3389/fdmed.2023.1120394 [25]	Review
3	Adelfio M, Ghezzi CE. Long-Term In Vitro Culture Systems to Study Human Microbiome. ACS Biomater Sci Eng. 2022 Nov 14;8(11):4613–4617. https://doi.org/10.1021/acsbiomaterials.1c01380. Epub 2022 Mar 24. PMID: 35324141; PMCID: PMC9508280. [73]	Review
4	Franca CM, Balbinot GS, Cunha D, Saboia VPA, Ferracane J, Bertassoni LE. In-vitro models of biocompatibility testing for restorative dental materials: From 2D cultures to organs on-a-chip. Acta Biomater. 2022 Sep 15;150:58–66. https://doi.org/10.1016/j.actbio.2022.07.060. Epub 2022 Aug 3. PMID: 35933103; PMCID: PMC9814917. [43]	Review
5	Regmi S, Poudel C, Adhikari R, Luo KQ. Applications of Microfluidics and Organ-on-a-Chip in Cancer Research. Biosensors (Basel). 2022 Jun 27;12(7):459. https://doi.org/10.3390/bios12070459. PMID: 35884262; PMCID: PMC9313151. [59]	Review
6	Rawas-Qalaji M, Cagliani R, Al-Hashimi N, Al-Dabbagh R, Al-Dabbagh A, Hussain Z. Microfluidics in drug delivery: review of methods and applications. Pharm Dev Technol. 2023 Jan;28(1):61–77. https://doi.org/10.1080/10837450.2022.2162543. Epub 2023 Jan 2. PMID: 36592376. [74]	Review
7	Soares DG, Bordini EAF, Swanson WB, de Souza Costa CA, Bottino MC. Platform technologies for regenerative endodontics from multifunctional biomaterials to tooth-on-a-chip strategies. Clin Oral Investig. 2021 Aug;25(8):4749–4779. https://doi.org/10.1007/s00784-021-04013-4. Epub 2021 Jun 28. PMID: 34181097; PMCID: PMC8546585. [75]	Review
8	Pagella P, Cordiale A, Marconi GD, Trubiani O, Rasponi M, Mitsiadis TA. Bioengineered tooth emulation systems for regenerative and pharmacological purposes. Eur Cell Mater. 2021 May 10;41:502–516. https://doi.org/10.22203/eCM.v041a32. PMID: 33970477. [42]	Review
9	Bertassoni LE. Progress and Challenges in Microengineering the Dental Pulp Vascular Microenvironment. J Endod. 2020 Sep;46(9S):S90-S100. https://doi.org/10.1016/j.joen.2020.06.033. PMID: 32950200; PMCID: PMC9924144. [44]	Review
10	Nashimoto Y, Hori T, Ostrovidov S, Katagiri S, Kaji H. Engineering Oral Microenvironments Using Microphysiological Systems. Sensors and Materials, Vol. 35, No. 4 (2023) 1293–1299 1293. https://doi.org/10.18494/SAM4164 [76]	Review
11	Farshidfar, N., Assar, S., Amiri, M.A. et al. The feasible application of microfluidic tissue/organ-on-a-chip as an impersonator of oral tissues and organs: a direction for future research. *Bio-des. Manuf.* 6, 478–506 (2023). https://doi.org/10.1007/s42242-023-00235-5 [77]	Review

**Table 7:** The 39 original studies included in the qualitative analysis.

Nr.	Reference	Topic
1	Kundu A, McCoy L, Azim N, Nguyen H, Didier CM, Ausaf T, Sharma AD, Curley JL, Moore MJ, Rajaraman S. Fabrication and Characterization of 3D Printed, 3D Microelectrode Arrays for Interfacing with a Peripheral Nerve-on-a-Chip. ACS Biomater Sci Eng. 2021 Jul 12;7(7):3018-3029. https://doi.org/10.1021/acsbiomaterials.0c01184. Epub 2020 Dec 10. PMID: 34275292. [40]	Tooth
2	França CM, Tahayeri A, Rodrigues NS, Ferdosian S, Puppin Rontani RM, Sereda G, Ferracane JL, Bertassoni LE. The tooth on-a-chip: a microphysiologic model system mimicking the biologic interface of the tooth with biomaterials. Lab Chip. 2020 Jan 21;20(2):405-413. https://doi.org/10.1039/c9lc00915a. Epub 2019 Dec 19. PMID: 31854401; PMCID: PMC7395925. [32]	Tooth
3	Hu S, Muniraj G, Mishra A, Hong K, Lum JL, Hong CHL, Rosa V, Sriram G. Characterization of silver diamine fluoride cytotoxicity using microfluidic tooth-on-a-chip and gingival equivalents. Dent Mater. 2022 Aug;38(8):1385-1394. https://doi.org/10.1016/j.dental.2022.06.025. Epub 2022 Jun 29. PMID: 35778310. [34]	Tooth
4	Rodrigues NS, França CM, Tahayeri A, Ren Z, Saboia VPA, Smith AJ, Ferracane JL, Koo H, Bertassoni LE. Biomaterial and Biofilm Interactions with the Pulp-Dentin Complex-on-a-Chip. J Dent Res. 2021 Sep;100(10):1136-1143. https://doi.org/10.1177/00220345211016429. Epub 2021 May 26. PMID: 34036838; PMCID: PMC8504857. [33]	Tooth
5	Kumar V, Kingsley D, Madhurakkat Perikamana S, Mogha P, Goodwin CR, Varghese S. Self-assembled innervated vasculature-on-a-chip to study nociception. Biofabrication. 2023 Apr 13;15(3):10.1088/1758-5090/acc904. https://doi.org/10.1088/1758-5090/acc904. PMID: 36996841; PMCID: PMC10152403. [39]	Tooth
6	Kramer L, Nguyen HT, Jacobs E, McCoy L, Curley JL, Sharma AD, Moore MJ. Modeling chemotherapy-induced peripheral neuropathy using a Nerve-on-a-chip microphysiological system. ALTEX. 2020;37(3):350-364. https://doi.org/10.14573/altex.2001181. Epub 2020 May 7. PMID: 32388569. [41]	Tooth
7	Pagella P, Neto E, Jiménez-Rojo L, Lamghari M, Mitsiadis TA. Microfluidics co-culture systems for studying tooth innervation. Front Physiol. 2014 Aug 25;5:326. https://doi.org/10.3389/fphys.2014.00326. [38]	Tooth
8	Zhang L, Han Y, Chen Q, Dissanayaka WL. Sema4D-plexin-B1 signaling in recruiting dental stem cells for vascular stabilization on a microfluidic platform. Lab Chip. 2022 Nov 22;22(23):4632-4644. https://doi.org/10.1039/d2lc00632d. PMID: 36331411. [37]	Tooth
9	Kim S, Lee H, Chung M, Jeon NL. Engineering of functional, perfusable 3D microvascular networks-on-a-chip. Lab Chip. 2013 Apr 21;13(8):1489-500. https://doi.org/10.1039/c3lc41320a. Erratum in: Lab Chip. 2013 Dec 21;13(24):4891. PMID: 23440068. [36]	Tooth
10	Qi Y, Zou T, Dissanayaka WL, Wong HM, Bertassoni LE, Zhang C. Fabrication of Tapered Fluidic Microchannels Conducive to Angiogenic Sprouting within Gelatin Methacryloyl Hydrogels. J Endod. 2021 Jan;47(1):52-61. https://doi.org/10.1016/j.joen.2020.08.026. Epub 2020 Oct 9. PMID: 33045266. [35]	Tooth
11	Niu L, Zhang H, Liu Y, Wang Y, Li A, Liu R, Zou R, Yang Q. Microfluidic Chip for Odontoblasts in Vitro. ACS Biomater Sci Eng. 2019 Sep 9;5(9):4844-4851. https://doi.org/10.1021/acsbiomaterials.9b00743. Epub 2019 Aug 1. PMID: 33448827. [31]	Tooth
12	Kang, KJ., Ju, S.M., Jang, YJ. et al. Indirect co-culture of stem cells from human exfoliated deciduous teeth and oral cells in a microfluidic platform. Tissue Eng Regen Med 13, 428–436 (2016). https://doi.org/10.1007/s13770-016-0005-2 [15]	Tooth
13	Tang PC, Eriksson O, Sjögren J, Fatsis-Kavalopoulos N, Kreuger J, Andersson DI. A Microfluidic Chip for Studies of the Dynamics of Antibiotic Resistance Selection in Bacterial Biofilms. Front Cell Infect Microbiol. 2022 May 10;12:896149. https://doi.org/10.3389/fcimb.2022.896149. PMID: 35619647; PMCID: PMC9128571. [29]	Tooth
14	Kristensen MF, Leonhardt D, Neland MLB, Schlafer S. A 3D printed microfluidic flow-cell for microscopy analysis of in situ-grown biofilms. J Microbiol Methods. 2020 Apr;171:105876. https://doi.org/10.1016/j.mimet.2020.105876. Epub 2020 Feb 19. PMID: 32087186. [30]	Tooth
15	Rath H, Stumpp SN, Stiesch M. Development of a flow chamber system for the reproducible in vitro analysis of biofilm formation on implant materials. PLoS One. 2017 Feb 10;12(2):e0172095. https://doi.org/10.1371/journal.pone.0172095. PMID: 28187188; PMCID: PMC5302373. [21]	Tooth
16	Lam RH, Cui X, Guo W, Thorsen T. High-throughput dental biofilm growth analysis for multiparametric microenvironmental biochemical conditions using microfluidics. Lab Chip. 2016 Apr 26;16(9):1652-62. https://doi.org/10.1039/c6lc00072j. PMID: 27045372. [16]	Tooth
17	Straub H, Eberl L, Zinn M, Rossi RM, Maniura-Weber K, Ren Q. A microfluidic platform for in situ investigation of biofilm formation and its treatment under controlled conditions. J Nanobiotechnology. 2020 Nov 11;18(1):166. https://doi.org/10.1186/s12951-020-00724-0. PMID: 33176791; PMCID: PMC7661213. [28]	Tooth
18	Gashti MP, Asselin J, Barbeau J, Boudreau D, Greener J. A microfluidic platform with pH imaging for chemical and hydrodynamic stimulation of intact oral biofilms. Lab Chip. 2016 Apr 21;16(8):1412-9. https://doi.org/10.1039/c5lc01540e. PMID: 26956837. [17]	Tooth
19	L. Jin, T. Tian, D. Liu, H. Mao and H. Liu, “·Reconstituting Organ-Level Periodontal Soft Tissue-on-a-chip,” 2021 21st International Conference on Solid-State Sensors, Actuators and Microsystems (Transducers), Orlando, FL, USA, 2021, pp. 707-710, https://doi.org/10.1109/Transducers50396.2021.9495506. [49]	Mucosa
20	Lee EJ, Kim Y, Salipante P, Kotula AP, Lipshutz S, Graves DT, Alimperti S. Mechanical Regulation of Oral Epithelial Barrier Function. Bioengineering (Basel). 2023 Apr 25;10(5):517. https://doi.org/10.3390/bioengineering10050517. PMID: 37237587; PMCID: PMC10215350. [48]	Mucosa
21	Makkar H, Zhou Y, Tan KS, Lim CT, Sriram G. Modeling Crevicular Fluid Flow and Host-Oral Microbiome Interactions in a Gingival Crevice-on-Chip. Adv Healthc Mater. 2023 Jan;12(6):e2202376. https://doi.org/10.1002/adhm.202202376. Epub 2022 Nov 28. PMID: 36398428. [19]	Mucosa
22	Sharifi F, Htwe SS, Righi M, Liu H, Pietralunga A, Yesil-Celiktas O, Maharjan S, Cha BH, Shin SR, Dokmeci MR, Vrana NE, Ghaemmaghami AM, Khademhosseini A, Zhang YS. A Foreign Body Response-on-a-Chip Platform. Adv Healthc Mater. 2019 Feb;8(4):e1801425. https://doi.org/10.1002/adhm.201801425. Epub 2019 Jan 29. PMID: 30694616; PMCID: PMC6398437. [47]	Mucosa
23	Rahimi C, Rahimi B, Padova D, Rooholghodos SA, Bienek DR, Luo X, Kaufman G, Raub CB. Oral mucosa-on-a-chip to assess layer-specific responses to bacteria and dental materials. Biomicrofluidics. 2018 Sep 26;12(5):054106. https://doi.org/10.1063/1.5048938. PMID: 30310527; PMCID: PMC6158033. [23]	Mucosa
24	Ly KL, Rooholghodos SA, Rahimi C, Rahimi B, Bienek DR, Kaufman G, Raub CB, Luo X. An Oral-mucosa-on-a-chip sensitively evaluates cell responses to dental monomers. Biomed Microdevices. 2021 Jan 11;23(1):7. https://doi.org/10.1007/s10544-021-00543-6. PMID: 33426594; PMCID: PMC8344876. [50]	Mucosa
25	Vurat MT, Şeker Ş, Lalegül-Ülker Ö, Parmaksiz M, Elçin AE, Elçin YM. Development of a multicellular 3D-bioprinted microtissue model of human periodontal ligament-alveolar bone biointerface: Towards a pre-clinical model of periodontal diseases and personalized periodontal tissue engineering. Genes Dis. 2020 Nov 28;9(4):1008-1023. https://doi.org/10.1016/j.gendis.2020.11.011. PMID: 35685479; PMCID: PMC9170773. [53]	Bone
26	Nasello G, Alamán-Díez P, Schiavi J, Pérez MÁ, McNamara L, García-Aznar JM. Primary Human Osteoblasts Cultured in a 3D Microenvironment Create a Unique Representative Model of Their Differentiation Into Osteocytes. Front Bioeng Biotechnol. 2020 Apr 24;8:336. https://doi.org/10.3389/fbioe.2020.00336. PMID: 32391343; PMCID: PMC7193048. [54]	Bone
27	Perottoni S, Neto NGB, Di Nitto C, Dmitriev RI, Raimondi MT, Monaghan MG. Intracellular label-free detection of mesenchymal stem cell metabolism within a perivascular niche-on-a-chip. Lab Chip. 2021 Apr 7;21(7):1395-1408. https://doi.org/10.1039/d0lc01034k. Epub 2021 Feb 19. PMID: 33605282. [52]	Bone
28	Jeon JS, Bersini S, Whisler JA, Chen MB, Dubini G, Charest JL, Moretti M, Kamm RD. Generation of 3D functional microvascular networks with human mesenchymal stem cells in microfluidic systems. Integr Biol (Camb). 2014 May;6(5):555-63. https://doi.org/10.1039/c3ib40267c. PMID: 24676392; PMCID: PMC4307755. [51]	Bone
29	Atif AR, Pujari-Palmer M, Tenje M, Mestres G. A microfluidics-based method for culturing osteoblasts on biomimetic hydroxyapatite. Acta Biomater. 2021 Jun;127:327-337. https://doi.org/10.1016/j.actbio.2021.03.046. Epub 2021 Mar 27. PMID: 33785452. [55]	Bone
30	Middleton K, Al-Dujaili S, Mei X, Günther A, You L. Microfluidic co-culture platform for investigating osteocyte-osteoclast signalling during fluid shear stress mechanostimulation. J Biomech. 2017 Jul 5;59:35-42. https://doi.org/10.1016/j.jbiomech.2017.05.012. Epub 2017 May 18. PMID: 28552413. [56]	Bone
31	Song Y, Uchida H, Sharipol A, Piraino L, Mereness JA, Ingalls MH, Rebhahn J, Newlands SD, DeLouise LA, Ovitt CE, Benoit DSW. Development of a functional salivary gland tissue chip with potential for high-content drug screening. Commun Biol. 2021 Mar 19;4(1):361. https://doi.org/10.1038/s42003-021-01876-x. Erratum in: Commun Biol. 2021 Apr 30;4(1):533. Erratum in: Commun Biol. 2022 Mar 30;5(1):315. PMID: 33742114; PMCID: PMC7979686. [70]	Salivary Glands
32	Yin Y, Vázquez-Rosado EJ, Wu D, Viswananthan V, Farach A, Farach-Carson MC, Harrington DA. Microfluidic coaxial 3D bioprinting of cell-laden microfibers and microtubes for salivary gland tissue engineering. Biomater Adv. 2023 Aug 14;154:213588. https://doi.org/10.1016/j.bioadv.2023.213588. Epub ahead of print. PMID: 37634337. [18]	Salivary Glands
33	Nguyen HQ, Seo TS. A 3D printed size-tunable flow-focusing droplet microdevice to produce cell-laden hydrogel microspheres. Anal Chim Acta. 2022 Feb 1;1192:339344. https://doi.org/10.1016/j.aca.2021.339344. Epub 2021 Dec 7. PMID: 35057943. [60]	Carcinoma
34	Pagella P, Catón J, Meisel CT, Mitsiadis TA. Ameloblastomas Exhibit Stem Cell Potential, Possess Neurotrophic Properties, and Establish Connections with Trigeminal Neurons. Cells. 2020 Mar 6;9(3):644. https://doi.org/10.3390/cells9030644. PMID: 32155948; PMCID: PMC7140461. [62]	Carcinoma
35	Kong J, Luo Y, Jin D, An F, Zhang W, Liu L, Li J, Fang S, Li X, Yang X, Lin B, Liu T. A novel microfluidic model can mimic organ-specific metastasis of circulating tumor cells. Oncotarget. 2016 Nov 29;7(48):78421-78432. https://doi.org/10.18632/oncotarget.9382. PMID: 27191997; PMCID: PMC5346650. [63]	Carcinoma
36	Liu, Lilu and Xie, Zhaorong and Zhang, Wenyuan and Fang, Shimeng and Kong, Jing and Jin, Dong and Li, Jiao and Li, Xiaojie and Yang, Xuesong and Luo, Yong and Lin, Bingcheng and Liu, Tingjiao. Biomimetic tumor-induced angiogenesis and anti-angiogenic therapy in a microfluidic model”, RSC Adv. 2016”, 6, 42, 35248-35256”, doi ==“10.1039/C6RA05645H” [20]	Carcinoma
37	Zoupanou S, Volpe A, Primiceri E, Gaudiuso C, Ancona A, Ferrara F, Chiriacò MS. SMILE Platform: An Innovative Microfluidic Approach for On-Chip Sample Manipulation and Analysis in Oral Cancer Diagnosis. Micromachines (Basel). 2021 Jul 27;12(8):885. https://doi.org/10.3390/mi12080885. PMID: 34442507; PMCID: PMC8401059. [61]	Carcinoma
38	Li J, Jia Z, Kong J, Zhang F, Fang S, Li X, Li W, Yang X, Luo Y, Lin B, Liu T. Carcinoma-Associated Fibroblasts Lead the Invasion of Salivary Gland Adenoid Cystic Carcinoma Cells by Creating an Invasive Track. PLoS One. 2016 Mar 8;11(3):e0150247. https://doi.org/10.1371/journal.pone.0150247. PMID: 26954362; PMCID: PMC4782997. [64]	Carcinoma
39	Al-Samadi A, Poor B, Tuomainen K, Liu V, Hyytiäinen A, Suleymanova I, Mesimaki K, Wilkman T, Mäkitie A, Saavalainen P, Salo T. In vitro humanized 3D microfluidic chip for testing personalized immunotherapeutics for head and neck cancer patients. Exp Cell Res. 2019 Oct 15;383(2):111508. https://doi.org/10.1016/j.yexcr.2019.111508. Epub 2019 Jul 26. PMID: 31356815. [65]	Carcinoma

## Data Availability

Not applicable.
